# Association between diabetes self‐management education attendance, hospital admissions and mortality in type 2 diabetes: A cohort analysis protocol

**DOI:** 10.1111/dom.16257

**Published:** 2025-02-19

**Authors:** Gemma A. Lewis, David M. Hughes, Greg Irving, John Wilding, Kevin Hardy

**Affiliations:** ^1^ Department of Diabetes and Endocrinology St Helens Hospital, Mersey and West Lancashire Teaching Hospitals NHS Trust St Helens UK; ^2^ Institute of Life Course and Medical Sciences University of Liverpool Liverpool UK; ^3^ Department of Health Data Science, Institute of Population Health University of Liverpool Liverpool UK; ^4^ Health Research Institute Edge Hill University Ormskirk UK

**Keywords:** cardiovascular disease, cohort study, database research, diabetes complications, observational study, type 2 diabetes

## Abstract

**Introduction:**

Type 2 diabetes is associated with excess hospital admissions and increased mortality. Structured diabetes self‐management education (DSME) is recommended internationally and is associated with improved self‐management skills, well‐being and minor improvements in glycated haemoglobin (HBA1c), but does it reduce hospital admissions or prevent premature mortality? Our aim is to examine the relationship between DSME attendance, hospitalisations, mortality and 3‐point major adverse cardiovascular events (MACE) in people with type 2 diabetes to inform future healthcare policy and diabetes care.

**Methods and Analysis:**

This protocol details a 10‐year retrospective open cohort study of patients aged over 18 years old who have a clinical diagnosis of type 2 diabetes and were registered to an English GP practice from 29 March 2011 to 29 March 2021 and have attended DSME. Patients in the '*ever*' cohort will be matched at baseline for age, sex, age at diagnosis and diabetes duration, to those who have '*never*' attended DSME. Data will be identified via the UK Clinical Practice Research Datalink and linked to Hospital Episode Statistics Admitted Patient Care data, Office for National Statistics death registrations and patient Index of Multiple Deprivation deciles. Patients will be followed‐up through serial cross‐sections. Multiple imputation will be considered to manage covariates where data are >12‐months from baseline or are not expected to be missing at random. Cox proportional hazard regression and time to event modelling adjusted a priori for cofounding during multivariate analysis will be used.

**Ethics and Dissemination:**

This study was approved by CPRD (24_003744). Study findings will be disseminated through peer‐reviewed publications and international conferences.

## 1 INTRODUCTION

Diabetes is a complex condition leading to disability and premature mortality with 7000 excess deaths in the United Kingdom in 2022.^1^ The International Diabetes Federation estimates 537 million people are living with diabetes globally^2^ of whom approximately 4.3 million reside in the United Kingdom.^3^ Over 90% have type 2 diabetes, with an increased prevalence in areas with high deprivation, poor healthcare access, poorer housing, reduced finances and lower educational attainment.^3^

Diabetes self‐management education (DSME), which includes support for healthy food and lifestyle choices, is fundamental to achieving good glycaemic control and is recommended internationally to help prevent diabetes‐related micro‐ and macrovascular complications.^4^, ^5^ Indeed, early delivery of DSME with timely pharmacotherapy may be particularly beneficial through the so‐called *legacy effect*.^6^, ^7^

Despite the National Institute for Health and Care Excellence (NICE),^5^ American Diabetes Association (ADA) and the European Association for the Study of Diabetes (EASD) stating that DSME is the foundation for successful diabetes management and prevention of metabolic comorbidities,^4^ the UK National Diabetes Audit suggests of 71% of people with type 2 diabetes offered DSME, only 7% attend.^8^

Approximately 20% of all UK hospital beds are currently occupied by people with diabetes and the number is expected to rise to 25% by 2030,^9^ most (92%) are admitted for other conditions and illnesses.^10^, ^11^

Of studies examining the impact of DSME on hospital admissions (*n* = 15), wide variability exists in the completeness of outcome data, confounding variables and design details making direct comparisons challenging. Four studies found DSME significantly impacted hospitalisations.^12^, ^13^, ^14^, ^15^ Time to first hospitalisation was significantly increased (*p* = 0.002) in a cohort of 376 US patients with type 2 diabetes over a 2‐year follow‐up, however, groups were not matched at baseline.^12^ A large cohort study in Hong Kong concluded that all‐cause admission rates significantly reduced following DSME in the 2‐year follow‐up, including those who only attended one session, with substantial delays to first admission and reduced recurrent admissions after DSME.^13^ In Serbian patients with type 2 diabetes, rates of admission due to metabolic conditions significantly reduced (*p* = 0.001) in the 6 months after DSME^14^ but longer term follow‐up was not reported. In Austria, although DSME did not impact number of admissions, it significantly reduced length of hospital stay (*p* = 0.04)^15^ but prevalence of type 2 diabetes in Austria is lower than other developed countries and all participants were recruited from one large teaching hospital potentially reducing its generalisability.

In studies examining the impact of DSME on mortality, most (*n* = 28) cite mortality as a reason for drop‐out rather than examining mortality as a primary outcome measure,^13^, ^16^, ^17^, ^18^ and only two studies report significant improvements following DSME.^13^, ^16^ In a study from Hong Kong, attending one or more DSME sessions was associated with lower all‐cause mortality (HR:0.59, CI 95% 0.632–0.968, *p* = 0.024); however, differences existed between study groups,^13^ with similar findings described in a UK randomised control trial, where mortality in participants requiring ambulance transfer for severe hypoglycaemia was 33.3% in those that had attended DSME compared with 51.2% in those receiving standard care without DSME (HR 0.65, *p* = 0.025).^16^ DSME workshops for an elderly Argentinian type 2 cohort resulted in an 33% lower 6‐year crude mortality rate, but significance was lost in multivariate analysis showing only a weak (18%) association between DSME attendance and reduced mortality (HR 0.82; 95%CI: 0.61–1.08) but socio‐economic data was not reported as a confounder, and those in the non‐attendance group had an increased stroke prevalence, higher diastolic blood pressure and LDL‐C which could explain the increased mortality rate.^17^ This weak association between DSME attendance and mortality was also reported in an earlier study which simulated the long‐term cost‐effectiveness of DSME interventions over 20 years, however, results obtained in this simulated RCT may not be representative of diabetes care delivered outside of this type of clinical trial.^18^

In short, existing studies provide evidence that DSME improves well‐being, self‐management skills and short‐term blood sugar control (HbA1c), but crucially evidence of an impact of DSME on hospital admissions and premature mortality is limited and conflicting and has not been assessed in a real world setting on this scale.

## 2 AIMS AND OBJECTIVES

The aim of this large retrospective cohort study is to examine the impact of DSME attendance on all‐cause and cause‐specific hospital admissions and mortality in people with type 2 diabetes, utilising linked large UK national datasets to test the hypotheses that DSME attendance impacts all‐cause hospital admissions and mortality.

The specific objectives are to:

1. To describe the epidemiology of patients living with type 2 diabetes who have attended DSME by age, sex, ethnicity, deprivation levels (IMD), HbA1c and baseline Charlson Comorbidity Index in a representative sample of a large cohort of adults who were registered with an English GP between 2011 and 2021.
2. To examine the association between ever or never having attended DSME on all‐cause and cause‐specific hospital admissions, in adults with type 2 diabetes.
3. To examine the association between ever or never having attended DSME on all‐cause and cause‐specific mortality, including but not confined to in‐hospital mortality.
4. To examine the association between ever or never having attended DSME on major adverse cardiovascular events (3P‐MACE); CV death, non‐fatal myocardial infarction (MI) and non‐fatal stroke, in adults with type 2 diabetes.

## 3 METHODS AND ANALYSIS

This study will be conducted, analysed and reported in line with the STrengthening the Reporting of OBservational studies in Epidemiology (STROBE) checklist for cohort studies.^19^, ^20^

### 3.1 Study design

This 10‐year retrospective open cohort study^19^ will include adults over the age of 18 years^†^, 12 months from the first use of a clinically recorded diagnosis of type 2 diabetes or type 2 diabetes‐related encounter registered to an English GP practice between 29 March 2011 and 29 March 2021^‡^. Patients will be identified from the CPRD database using READ codes to run analyses of the agreed set of exposures and outcomes.

^†^Patients who turn 18 during the study period will be included from the point of turning 18.

‡At the time of CPRD approval for this protocol data linkage to Hospital Episode Statistics (HES) Admitted Patient Care (APC) data were restricted up to and including 29 March 2021.

### 3.2 Sample

Patients will be stratified into two groups dependent on their exposure to DSME. The primary cohort is those who have a READ code confirming they have ‘*ever’* received DSME (*n* = 21 298) (Table 1 lists the Med Code IDs for exposure to DSME). Guidelines recommend DSME for all patients newly diagnosed with type 2 diabetes.^5^ To allow sufficient time for exposure to DSME and to account for immortal time bias^21^ any hospitalisations or deaths within the first 12 months of diabetes diagnosis will be excluded in both ever and never DSME groups as it is likely that there will be a period during the 12 months in which patients have not had the opportunity to be exposed to the intervention.

**TABLE 1:** DSME Med Code IDs.

Med Code ID	Term
2 533 102 013	Attended diabetes structured education programme
5.32331E+14	Diabetes structured education programme completed
5.46001E+14	Attended XPERT diabetes structured education programme
5.46221E+14	DESMOND diabetes structured education programme completed
5.46291E+14	XPERT diabetes structured education programme completed
6.84397E+15	Attended diabetes structured education programme
8.09822E+15	Attended expert patient education versus routine treatment diabetes structured education programme
8.09824E+15	Attended XPERT (expert patient education versus routine treatment) diabetes structured education programme
8.09835E+15	Expert patient education versus routine treatment diabetes structured education programme completed
8.09837E+15	XPERT (expert patient education versus routine treatment) diabetes structured education programme completed
1.21156E+16	Remote diabetes structured education and support programme commenced
1.21156E+16	Remote diabetes structured education and support programme completed

### 3.3 Data selection

To minimise the impact of incorrect information or flawed subject selection the ‘*ever’* cohort will be matched at baseline to patients who have ‘*never’* received DSME (*n* = 584 312). Age,^22^, ^23^ age at diagnosis and diabetes duration^22^, ^24^, ^25^, ^26^ are the most important predictors of one‐year mortality in type 2 diabetes.^27^ Every decade of earlier diabetes diagnosis is associated with 3–4 years of lower life expectancy.^24^

Cohort analysis, systematic review and meta‐analysis have considered the association between diabetes and sex, demonstrating that women generally have a higher risk of coronary heart disease and all‐cause mortality (58%) compared to men and a 13%–17% greater risk of all‐cause mortality.^28^, ^29^ Moreover, the additional likelihood of developing cancer and the higher risk of premature mortality from having diabetes is more pronounced in women.^30^

Patients will therefore be matched at baseline for age ± 5 years, age at diagnosis ±5 years, diabetes duration ±1 year and sex who are present in the dataset from the same start year or earlier.

This study will adopt an intention‐to‐treat analysis, with patients contributing only to the original exposure group in which they have been assigned, irrespective of when they receive DSME. Baseline for the ‘ever’ group and their matched controls will be the date of DSME intervention. Patients will remain exposed until they are censored by death, leave the GP practice, leave a GP practice which contributes to CPRD data or end of the study period (29 March 2021).

### 3.4 Data processing and analysis

To increase the efficiency of clinical trials researchers embarking on prospective longitudinal studies will often include restrictive eligibility criteria to restrict potential mortality from existing comorbid conditions.^22^ Such restrictions in retrospective studies limit generalisability of the findings and result in significant loss of individuals included in the study. An alternative approach introduced by Charlson^22^ classifies patients with comorbid disease according to their risk of death from disease at entry to a study, allowing researchers to classify the risk of one‐year and 10‐year mortality.^22^, ^31^ Charlson Comorbidity Index (CCI) can be classified as mild (1–2), moderate (3–4) and severe (≥5).^25^ With each increased level of the comorbidity index, there is a stepwise increase in the cumulative mortality attributable to comorbid disease.^22^ CCI will be calculated from CPRD Aurum to predict a patient's 10‐year risk of mortality and managed as a confounding factor during multivariate analysis.

Not all aspects of health data are captured in CPRD, for example, socio‐economic demographics which are an independent effect modifier contributing to increased mortality in people with type 2 diabetes, with mortality risk increased in those in the most deprived neighbourhoods by as much as 11% per quintile of deprivation where the person also had a diabetic foot ulcer.^32^ Linkage to patient level indices of multiple deprivation (IMD) deciles will overcome this. IMD will be managed as a cofounding factor during multivariate analysis and this limitation will need to be addressed fully in our discussion.

We will use primary care demographic data and READ codes for CPRD Aurum and laboratory tests to confirm main potential confounders at baseline. These include age, sex, time since first use of diagnosis code (as a proxy for diabetes duration and age at diagnosis in years), multimorbidity (CCI), BMI, smoking status and laboratory results for HbA1c, systolic blood pressure (SBP) and total cholesterol. Ethnicity and patient level IMD will be included following linkages with relevant health datasets.

**FIGURE 1:**
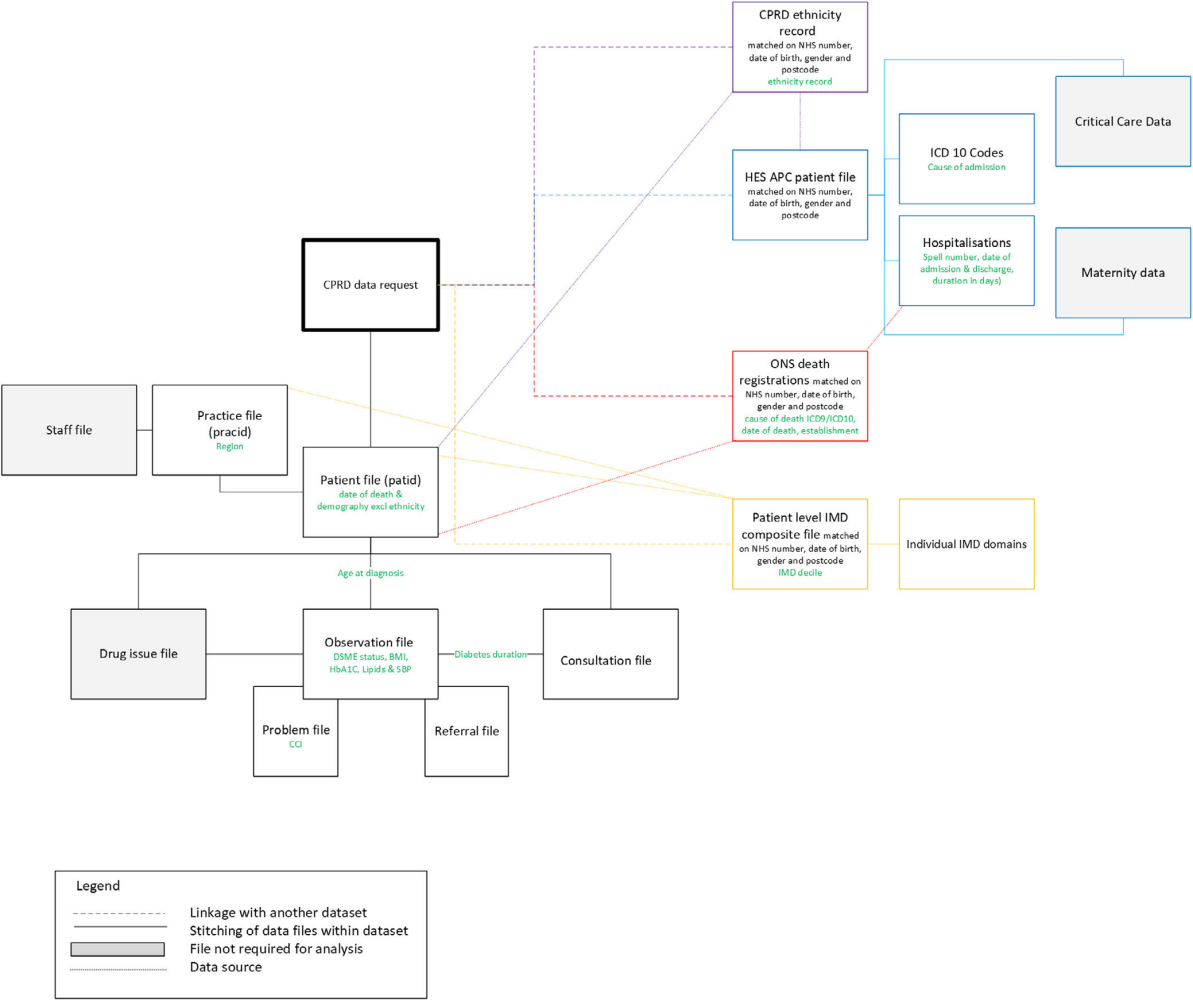
Stitching of data and linkage of datasets.^35^, ^36^

The CPRD dataset will be cleaned and checked for completeness prior to linkage with patient level indices of multiple deprivation (IMD), Hospital Episode Statistics (HES) Admitted Patient Care (APC) and Office for National Statistics (ONS) death registry datasets. Figure 1 demonstrates a visual representation of how data are stitched together and linked with other datasets.

Following data linkage to the HES APC dataset, ICD codes for 17 cause‐specific mutually exclusive admission clusters (Appendix 1) will be extracted as identified in a recent epidemiological analysis for people with diabetes in England^11^: ischaemic heart disease, stroke, acute myocardial infarction (MI), diabetes‐related cancers, non‐diabetes related cancers, hyperglycaemic crisis, major amputations, minor amputations, skin and bone infections, respiratory infections, kidney infections, sepsis, respiratory disease (non‐infectious, non‐cancerous), renal disease, chronic kidney disease, liver disease.

More than 50% of deaths in the United Kingdom do not occur in hospital, with ONS mortality data considered the gold standard for mortality records.^33^ Following data linkage with the ONS national death registry, cause‐specific mortality across 12 mutually exclusive clusters (Appendix 2) will be extracted as identified in a recent epidemiological study identifying causes of death in patients with diabetes in England^34^: ischaemic heart disease, stroke, other circulatory, other cancer, diabetes‐related cancer, dementia, diabetes, liver, renal, respiratory, other, injuries. In line with current reconciling guidelines date of death and cause of death will be taken from CPRD to enable linkage for the 25.11% of patients in England that are ineligible for linkage with ONS.^33^

The following recommendations will be applied when linking data from CPRD to ONS mortality data.^33^

1. If only CPRD date of death is present, this can be used as a reliable piece of information and has been previously validated.
2. If only ONS mortality information is present, its date and causes of death can be used if the linkage rank is ≤2 (Table 2) and the date of death is within the linkage period being examined.
3. If both CPRD and ONS dates of death are available, ONS will be used for the date of death and cause of death when the match rank is ≤2 and the date of death is within the linkage period being examined.
4. If both CPRD and ONS are present and ONS date of death is outside the linkage period, or the match is ≥3 the CPRD date will be used to validate ONS. If the dates coincide ONS date of death and cause of death can be used. If not only CPRD date will be used.

**TABLE 2:** National Health Service (NHS) digital 8‐step linkage algorithm.^33^

Step	NHS number	Date of birth	Gender	Postcode
1	Exact	Exact	Exact	Exact
2	Exact	Exact	Exact	
3	Exact	Partial	Exact	Exact
4	Exact	Partial	Exact	
5	Exact			Exact
6		Exact	Exact	Exact
7		Exact	Exact	Exact
8	Exact			

### 3.5 Study size

The study sample includes all patients in CPRD Aurum available during the study period who meet the inclusion criteria. A feasibility search on Aurum identified people meeting the study inclusion criteria (*n* = 605 710). A priori calculation of the sample size whilst presenting some uncertainty cannot be determined beforehand as the sample size will be reduced by inclusion of confounding variables and exclusion of some individuals.^19^ The sample size included in this study from feasibility counts is far larger than usually considered in hypothesis testing situations such as those proposed in this study. This gives us confidence we have sufficient sample size in this study for a reliable assessment of the research questions. Nevertheless, as an example, the 21 398 ‘*ever*’ patients and 584 312 ‘*never*’ patients identified would be sufficient to detect a difference in the proportion hospitalised (main outcomes) of approximately 1.9% with a power of 80% and a significance level of 5%.

### 3.6 Statistical methods

Baseline characteristics of participants will be summarised using descriptive statistics (mean, median, interquartile range and standard deviations as appropriate). We will also summarise total number of hospital admissions and length of stay by age, age at time of diabetes diagnosis, diabetes duration, ethnicity and IMD decile.

The primary cohort is those who have ‘*ever*’ received DSME in each of the 10 years in the study to identify hospital admissions and cause of death during follow‐up. We will additionally have a matched diabetes cohort who have ‘*never’* received DSME to provide comparison (rate ratios).

An initial crude analysis will compare the proportion of hospitalisations and deaths between *ever* and *never* DSME groups. Chi‐squared tests will be used to compare these proportions. Similar tests will be used for subgroup analysis and alternative outcomes (cause‐specific hospitalisations and mortality).

Number of hospital admissions will be calculated to estimate relative risk of admissions and mortality by all‐cause and cause‐specific using a mixed Poisson model. Should the data be over dispersed a negative binomial mixed model will be adopted.

A Cox proportional‐hazards model will be used to measure time to event for first hospital admissions and mortality with the proportional hazards assumption checked using Kaplan—Meier curve plots and Schoenfeld residuals tests. If the proportional hazards assumption does not hold, then alternatives such as restricted mean survival times models will be used.

Baseline covariates will be reported among the ‘ever’ and ‘never’ groups, and a statistical test will be applied to see whether these differ significantly. These will be examined in multivariate models. We will address potential issues with confounding by indication firstly within the study design in which individuals in the ever group will be matched at baseline for age, sex, diabetes duration and age at diagnosis to individuals in the never group. All models will be adjusted a priori for CCI, IMD, HbA1c, BMI, smoking status, lipid level and SBP during multivariate analysis.

Statistical analysis will be conducted using R version 4.4.1. Alpha level will be set at 5% for statistical significance.

### 3.7 Plans for addressing missing data

Assessment of data quality and completeness in CPRD Aurum suggests that correctness of type 2 diabetes is high (94%–98%) indicating that a diagnosis code if found is likely to be correct and there are likely very few patients with type 2 diabetes in the absence of a code.^37^ In our primary analysis, we will use a complete‐case approach based on the main potential confounders listed. We will conduct secondary analyses using complete cases for the full list of potential confounders, including those expected to have a high proportion of missing data which we do not expect to be missing at random.

CCI will be used to calculate an individual's 10‐year risk of mortality. As it cannot be assumed that covariate measurements (CCI, ever or never attended DSME, diagnostic codes) are missing at random we will not use multiple imputation for these measures.

Data will be recorded as missing where there is no record within 12 months of baseline. Depending on completeness of data a decision will be made on whether to use multiple imputation to manage covariates. As HbA1c, lipids and BP measures are amongst the nine key care processes^5^ completed within primary care every 12 months and linked to the GP Quality Outcome Framework, it is likely these are well completed with the dataset.

## 4 DISCUSSION

The persistent increased gap in the rate of hospital admissions and premature mortality rates for people living with diabetes is well documented. Fundamental to achieving good glycaemic control and reduced diabetes associated micro‐ and macrovascular complications is internationally recommended DSME. Existing literature provides evidence that DSME improves quality of life, self‐management skills and short term HbA1c, but persuasive evidence that DSME impacts hospital admissions or premature mortality for people living with type 2 diabetes is lacking.

Heterogeneity of DSME programmes make comparison challenging.^38^, ^39^, ^40^, ^41^ Although accreditation and regulation of DSME programmes is considered as good practice it is not compulsory and differences in the mode, session content, duration and quality assurance of programmes are common. This study will not examine the heterogeneity of different DSME programmes. All programmes reported using relevant Med Code ID codes (presented in Table 1) will be included. Further studies will be required to examine the relationship between DSME variance and outcomes.

Existing UK practice for DSME makes retrospective cohort studies such as ours challenging. DSME attendance is defined as having engaged with 10% of a course's content and completion is defined as having engaged with 60% of a programme's content,^42^, ^43^, ^44^ despite evidence of benefit largely being derived from 100% engagement. Whilst we recognise limitations this poses for retrospective cohort analysis, as this is the nationally accepted coding and reporting model for DSME engagement, and as we are unable to presume missingness of data for DSME attendance in medical records, the existing model will be utilised to determine ‘ever’ and matched ‘never’ cohorts. Secondary analysis will examine differences between attended and completed codes. Further studies are required to analyse the impact of these nationally adopted metrics, which is beyond the scope of this study but is being examined elsewhere.^42^

This study addresses an important gap in the literature using a large real‐world dataset, representative of the general population, linked to HES APC, ONS death registrations, patient level IMD and ethnicity datasets. The design overcomes many of the limitations of previous studies and its scale and matching of patients at baseline allows us to examine the impact of attending DSME on all‐cause and cause‐specific hospital admissions and mortality in people with type 2 diabetes.

### 4.1 Limitations, generalisability and interpretation

The medical data available in CPRD are collected during clinical consultations rather than for research, thus the data may be limited by recording quality, and there may be variation in the consistency and accuracy of reporting and coding by GPs. This may introduce inaccuracy into the study findings.

Although CPRD data on diabetes diagnosis has a high level of completeness, not all patient groups or GP practices are captured, for example, private patients, prisoners or some residential home patients. Moreover, some patients chose to opt out of CPRD, and some are exception‐reported by GPs. Furthermore, CPRD data capture may be less complete for ethnicity and socio‐economic demographic data, but this can be largely overcome by data linkage.

This study is observational by nature and reverse causality cannot be excluded as a possible explanation for associations or inferences. We are reliant on the accuracy of ICD‐10 codes and SNOMED‐CT codes to define outcomes. These limitations may introduce bias to the study and will be fully acknowledged in the discussion. Although we will match groups at baseline and manage confounding a priori, potential residual confounding can never be completely eliminated, and causality would need to be confirmed in a randomised controlled trial.

This study has the potential to benefit patients by informing guidelines and clinical strategies for people living with type 2 diabetes.

## AUTHOR CONTRIBUTIONS

GL conceptualised the study. GL, KH, DH, JW, GI contributed to the design and review of the study protocol. GL drafted the manuscript. KH, DH and GI reviewed the manuscript. GL and KH approved the final version. The advisory team, which incorporates the supervisors KH, DH, JW, GI, will supervise the study and monitor data collection process, analysis and write‐up.

## FUNDING INFORMATION

No funding has been awarded for this study.

## CONFLICT OF INTEREST STATEMENT

GI is the National NIHR Research Delivery Network lead for General Practice. JW reports consultancy/advisory board work for the pharmaceutical industry contracted via the University of Liverpool (no personal payment) for Altimmune, AstraZeneca, Boehringer Ingelheim, Cytoki, Lilly, Napp, Novo Nordisk, Menarini, Pfizer, Rhythm Pharmaceuticals, Sanofi, Saniona, Tern, Shionogi and Ysopia; research grants for clinical trials from AstraZeneca and Novo Nordisk and personal honoraria/lecture fees from AstraZeneca, Boehringer Ingelheim, Medscape, Napp, Novo Nordisk and Rhythm. JW is past president of the World Obesity Federation, a member of the Association for the Study of Obesity, Diabetes UK, EASD, ADA, Society for Endocrinology and the Rank Prize Funds Nutrition Committee. From 2009 to 2024 he was national lead for the Metabolic and Endocrine Specialty Group of the UK NIHR Clinical Research Network. GL, DH and KH report no conflicts of interest.

## ETHICS STATEMENT

CPRD collects anonymised patient data from a network of GP practices across the United Kingdom which are linked to other health related data to provide a longitudinal, representative UK population health dataset. CPRD research data services are delivered by the UK Medicines and Healthcare products Regulatory Agency (MHRA) with support from the National Institute for Health and Care Research. Protocol approval was granted by CPRD (24_003744). Individual patient level consent was not required for this study as all data are deidentified.

### PEER REVIEW

The peer review history for this article is available at https://www.webofscience.com/api/gateway/wos/peer‐review/10.1111/dom.16257.

## Data Availability

Data sharing not applicable to this article as no datasets were generated or analysed during the writing of this protocol.

## APPENDIX A

**TABLE A1:** ICD‐10 codes for each underlying cause of hospitalisation and cause groupings [1].

Underlying causes	ICD‐10 Codes	Tier 1 cause groupings	Tier 2 Cause groupings
Malignant neoplasm of liver and intrahepatic bile ducts	C22	Cancers	DM‐related cancers
Malignant neoplasm of colon, rectosigmoid junction and rectum (i.e. colorectal)	C18, C19, C20	Cancers	DM‐related cancers
Malignant neoplasms of digestive organs except liver and intrahepatic bile ducts or colorectal	C15–C17, C21, C24, C26	Cancers	All other cancers
Malignant neoplasm of gallbladder	C23	Cancers	DM‐related cancers
Malignant neoplasm of pancreas	C25	Cancers	DM‐related cancers
Malignant neoplasms of lymphoid, haematopoietic and related tissue	C81–C96	Cancers	All other cancers
Malignant neoplasm of trachea, bronchus and lung	C33–C34	Cancers	All other cancers
Malignant neoplasm of prostate	C61	Cancers	All other cancers
Malignant neoplasm of breast	C50	Cancers	DM‐related cancers
Malignant neoplasm of cervix	C53	Cancers	All other cancers
Malignant neoplasm of corpus uteri	C54	Cancers	DM‐related cancers
All other neoplasms	C00–C14, C27–C32, C35–C49, C51–C52, C55–C60, C62–C80, C97–D48	Cancers	All other cancers
Respiratory	J00–J98, J19, J22–J99	Respiratory, renal & liver	Respiratory
Respiratory infection	J12–J18, J09–J12, J20–21, A15–A19	Infections	Respiratory infection
Diabetes	E10–E14, excluding E10.0, E10.1, E11.0, E11.1, E12.0, E12.1, E13.0, E13.1, E14.0, E14.1	Diabetes	Diabetes
Acute Myocardial Infarction	I21–I22	Vascular	Acute myocardial infarction
Other ischaemic heart disease & heart failure	I20, I23–I25, I50	Vascular	Ischaemic heart disease
Stroke (cerebrovascular)	I60–I69	Vascular	Stroke
Major Lower limb amputations	OPCS codes: 093–095, 098–099	Amputations	Major amputations
Minor lower limb Amputations	OPCS codes: 101, 104, 108–109, 111–112, 118–119	Amputations	Minor amputations
Hyperglycaemic crisis	E10.0, E10.1, E11.0, E11.1, E12.0, E12.1, E13.0, E13.1, E14.0, E14.1	Diabetes	Hyperglycaemic crisis
Renal disease	N00–N09, N12–N14, N17, N19–N28	Respiratory, renal & liver	Renal disease
ESRD	N18	Respiratory, renal & liver	Renal disease
Kidney infection	N10–11, N15–N16, N30, N39	Infections	Kidney infection
Liver disease	K70–77	Respiratory, renal & liver	Liver disease
Skin, soft tissue and bone infections	L03, L97, I96, M86, B35–49	Amputations	Skin and bone infections
Sepsis	A40–A41	Sepsis	Sepsis

^1^ Pearson‐Stuttard J, Cheng YJ, Bennett J, *et al*. Trends in leading causes of hospitalisation of adults with diabetes in England from 2003 to 2018: an epidemiological analysis of linked primary care records. *Lancet Diabetes Endocrinol*. 2022;10. DOI: 10.1016/S2213‐8587(21)00288‐6.

**TABLE A2:** ICD‐10 codes for each underlying cause of mortality and cause groupings [1].

Underlying causes	ICD‐10 Codes	Tier 1 cause groupings	Tier 2 Cause groupings
Malignant neoplasm of liver and intrahepatic bile ducts	C22	Cancers	DM‐related cancers
Malignant neoplasm of colon, rectosigmoid junction and rectum (i.e. colorectal)	C18, C19, C20	Cancers	DM‐related cancers
Malignant neoplasms of digestive organs except liver and intrahepatic bile ducts or colorectal	C15–C17, C21, C24, C26	Cancers	All other cancers
Malignant neoplasm of gallbladder	C23	Cancers	DM‐related cancers
Malignant neoplasm of pancreas	C25	Cancers	DM‐related cancers
Malignant neoplasms of lymphoid, haematopoietic and related tissue	C81–C96	Cancers	All other cancers
Malignant neoplasm of trachea, bronchus and lung	C33–C34	Cancers	All other cancers
Malignant neoplasm of prostate	C61	Cancers	All other cancers
Malignant neoplasm of breast	C50	Cancers	DM‐related cancers
Malignant neoplasm of cervix	C53	Cancers	All other cancers
Malignant neoplasm of corpus uteri	C54	Cancers	DM‐related cancers
All other neoplasms	C00–C14, C27–C32, C35–C49, C51–C52, C55–C60, C62–C80, C97–D48	Cancers	All other cancers
Respiratory	J00–J99	Other	Respiratory
Diabetes	E10–E14	Other	Diabetes
Ischaemic Heart Disease	I20–I25	Vascular	Ischaemic heart disease
Stroke (cerebrovascular)	I60–I69	Vascular	Stroke
Other circulatory	I00–I19, I26–I59, I70–I99	Vascular	Other circulatory
Renal disease	N00–N28	Other	Renal disease
Liver disease	K70–77	Other	Liver disease
Intentional injury – assault	X85–Y09, Y871	Other	Accidents
Intentional Injury – self harm	X60–X84, Y870	Other	Accidents
Intentional Injury – other intentional injury	Y35, Y36	Other	Accidents
Unintentional injury	V01–X59, Y40–Y86, Y88, Y89	Other	Accidents
Dementia and Alzheimer's	F00–F03, G30	Other	Dementia
All other ICD codes		Other	Other

^1^ Pearson‐Stuttard J, Bennett J, Cheng YJ, *et al*. Trends in predominant causes of death in individuals with and without diabetes in England from 2001 to 2018: an epidemiological analysis of linked primary care records. *Lancet Diabetes Endocrinol*. 2021;9. DOI: 10.1016/S2213‐8587(20)30431‐9.
